# Role of Hyperbaric Oxygenation Plus Hypofractionated Stereotactic Radiotherapy in Recurrent High-Grade Glioma

**DOI:** 10.3389/fonc.2021.643469

**Published:** 2021-03-30

**Authors:** Donatella Arpa, Elisabetta Parisi, Giulia Ghigi, Annalisa Cortesi, Pasquale Longobardi, Patrizia Cenni, Martina Pieri, Luca Tontini, Elisa Neri, Simona Micheletti, Francesca Ghetti, Manuela Monti, Flavia Foca, Anna Tesei, Chiara Arienti, Anna Sarnelli, Giovanni Martinelli, Antonio Romeo

**Affiliations:** ^1^ Radiotherapy Unit, IRCCS Istituto Romagnolo per lo Studio dei Tumori (IRST) “Dino Amadori”, Meldola, Italy; ^2^ Centro Iperbarico, Ravenna, Italy; ^3^ Neuroradiology Unit, “Santa Maria delle Croci” Hospital, Ravenna, Italy; ^4^ Unit of Biostatistics and Clinical Trials, IRCCS Istituto Romagnolo per lo Studio dei Tumori (IRST) “Dino Amadori”, Meldola, Italy; ^5^ Biosciences Laboratory, IRCCS Istituto Romagnolo per lo Studio dei Tumori (IRST) “Dino Amadori”, Meldola, Italy; ^6^ Medical Physics Unit, IRCCS Istituto Romagnolo per lo Studio dei Tumori (IRST) “Dino Amadori”, Meldola, Italy; ^7^ Scientific Directorate, IRCCS Istituto Romagnolo per lo Studio dei Tumori (IRST) “Dino Amadori”, Meldola, Italy

**Keywords:** recurrent high-grade glioma, hypofractionated stereotactic radiotherapy, hyperbaric oxygenation, TomoTherapy, re-irradiation

## Abstract

**Background:**

The presence of hypoxic cells in high-grade glioma (HGG) is one of major reasons for failure of local tumour control with radiotherapy (RT). The use of hyperbaric oxygen therapy (HBO) could help to overcome the problem of oxygen deficiency in poorly oxygenated regions of the tumour. We propose an innovative approach to improve the efficacy of hypofractionated stereotactic radiotherapy (HSRT) after HBO (HBO-RT) for the treatment of recurrent HGG (rHGG) and herein report the results of an *ad interim* analysis.

**Methods:**

We enrolled a preliminary cohort of 9 adult patients (aged >18 years) with a diagnosis of rHGG. HSRT was administered in daily 5-Gy fractions for 3-5 consecutive days a week. Each fraction was delivered up to maximum of 60 minutes after HBO.

**Results:**

Median follow-up from re-irradiation was 11.6 months (range: 3.2-11.6 months). The disease control rate (DCR) 3 months after HBO-RT was 55.5% (5 patients). Median progression-free survival (mPFS) for all patients was 5.2 months (95%CI: 1.34-NE), while 3-month and 6-month PFS was 55.5% (95%CI: 20.4-80.4) and 27.7% (95%CI: 4.4-59.1), respectively. Median overall survival (mOS) of HBO-RT was 10.7 months (95% CI: 7.7-NE). No acute or late neurologic toxicity >grade (G)2 was observed in 88.88% of patients. One patient developed G3 radionecrosis.

**Conclusions:**

HSRT delivered after HBO appears to be effective for the treatment of rHGG, it could represent an alternative, with low toxicity, to systemic therapies for patients who cannot or refuse to undergo such treatments.

**Clinical Trial Registration:**

www.ClinicalTrials.gov, identifier NCT 03411408.

## Introduction

High-grade gliomas (HGGs) represent the most malignant and most frequently encountered primary brain tumour in clinical neuro-oncology. Despite improvements in diagnostic and therapeutic strategies, the clinical prognosis for patients with HGG remains poor, with a median overall survival of <16 months. The majority of cases relapse within a year of diagnosis, and almost always at the initial site of disease (1). Life expectancy in this group is even poorer, with a median survival of around 6-11 months (2–6). Developing effective salvage treatments at recurrence is thus urgently needed to prolong overall survival (7). Hypoxia is thought to play a role in tumour development, angiogenesis and growth, and resistance to chemotherapy, antiangiogenic therapy and radiotherapy (RT) in a large number of human cancers (8, 9). Brain tumours, especially highly aggressive GBM with its necrotic tissue, are more likely to be affected by hypoxia. GBM is a highly vascularized tumour with a functionally inefficient microcirculation that may contribute to hypoxia and necrosis within a tumour (10–15). Several studies have reported that the median partial pressure of oxygen (PO2) of high-grade gliomas in patients under anaesthesia was approximately 5-7 mmHg, with a significant proportion of PO2 values <2.5 mmHg (16–19). The radiosensitivity of brain tumours could potentially be increased by performing hyperbaric oxygenation (HBO) before the RT session (20–25).

Recent studies suggest that the PO2 within tumours increases during HBO and is maintained for several minutes after the procedure (17, 26, 27). It is known that the cellular metabolism of malignant glioma is anaerobic, with the tumour exhibiting a lower oxygen consumption rate than normal white matter (28, 29). Thus, in contrast to normal brain tissue, the PO2 within the tumour decreases more slowly after decompression because of low oxygen consumption and poor blood flow to the tumour. It can thus be hypothesized that HBO before RT is capable of increasing the sensitivity of hypoxic tumour cells to treatment without increasing the damage to normal brain tissue (20, 30–32).

We propose an innovative approach to improve the efficacy of HSRT using image-guided helical TomoTherapy after HBO for the treatment of recurrent HGG (rHGG). Herein we report the results of an *ad interim* analysis.

## Materials and Methods

### Patient Eligibility

Adult patients **(**aged >18 years) with rHGG, as defined by RANO (Response Assessment for Neuro-Oncology) criteria (33), underwent HBO followed by re-irradiation (RE-RT). Main inclusion criteria are shown in **Table 1**. Exclusion criteria were as follows: radiotherapy ≤12 weeks prior to diagnosis of progression if the lesion was in the radiation field; b) cardiopulmonary disease (heart failure, bullous emphysema, pneumothorax, chronic obstructive pulmonary disease with hypercapnia sinusitis); and closed-angle glaucoma with ocular pressure >24 mmHg.

**Table 1:** Eligibility criteria.

• Male or female, aged >18 years • Karnofsky Performance Scale (KPS)> 60 • Imaging confirmation of first tumour progression or regrowth as defined by RANO criteria at least 12 weeks after completion of radiotherapy, unless the recurrence is outside the radiation field or has been histologically documented. • Recurrence after adjuvant treatment (surgery followed by radiotherapy and chemotherapy) • Adequate bone marrow, liver function and renal function measured by laboratory tests no more than 7 days before start of study treatment. • Participant is willing and able to give informed consent to take part in the study. • If female and of child-bearing potential, the patient must have a negative pregnancy test a maximum of 7 days before starting therapy.

RANO, Response Assessment in Neuro-Oncology.

### Study Design

This was a pilot study of Hypofractionated Stereotactic Radiotherapy (HSRT) using TomoTherapy. This trial provided 5 Gy/day for 3-5 consecutive days after daily HBO for the treatment of recurrent malignant high-grade glioma(rHGGs). The maximum time from completion of decompression to HRT was 60 min. The primary objective of this study was to evaluate the disease control rate (DCR) of treated patients. DCR was defined as the percentage of patients with rHGG who have achieved complete response, partial response and stable disease 3 months after HBO-RT. Secondary objectives were safety assessment (acute and late toxicity), OS and progression-free survival (PFS).

### HBO Therapy

HBO was administrated in a multiplace hyperbaric chamber according to the following schedule: ten min of compression with a fraction of inspired oxygen (*Fi*O2) >90% from 152 to 253 kilopascal, 60 min of *Fi*O2 >90% at 253 kilopascal (three breathing cycles in oxygen of 22 min each, with 2-minute intervals breathing air and 10 min of decompression with a *Fi*O2 >90% from 253 to 152 kilopascal. Following the HBO session, each patient underwent RT.

### Radiotherapy

#### Target Volume Definition

Computed tomography (CT) planning (Brilliance Big Bore CT Philips, Crowley, UK) was performed with a 1- to 3-mm slice thickness. Patients were placed in a supine position with arms close to their body and immobilized with a thermoplastic mask. A co-registration of volumetric CT and magnetic resonance imaging (MRI) sequences (1- to 3-mm slice thickness) was performed to define the targets and organs-at-risk (OAR). MRI sequences were performed using a 1.5-T with T1-weighted imaging, contrast-enhanced T1-weighted axial imaging with gadolinium (Gd-MRI), fluid-attenuated inversion recovery imaging (FLAIR), axial T2- weighted imaging, coronal T2-weighted imaging, DWI (diffusion-weighted imaging), dynamic susceptibility contrast-enhanced (DSC) and dynamic contrast-enhanced (DCE) perfusion. The planning treatment volume 1 (PTV1) was defined as the visible tumour on enhanced T1-MRI with a 1-mm margin expansion. In accordance with the neuroradiology team, another treatment volume (PTV FLAIR) was delineated to include the surrounding edema in cases where non-enhanced areas highlighted by increased T2-weighted FLAIR signal were evaluated as disease progression. OARs were identified as healthy brain, optic chiasm, optic nerves and brainstem.

#### Treatment Planning and Irradiation

**Figure 1:**
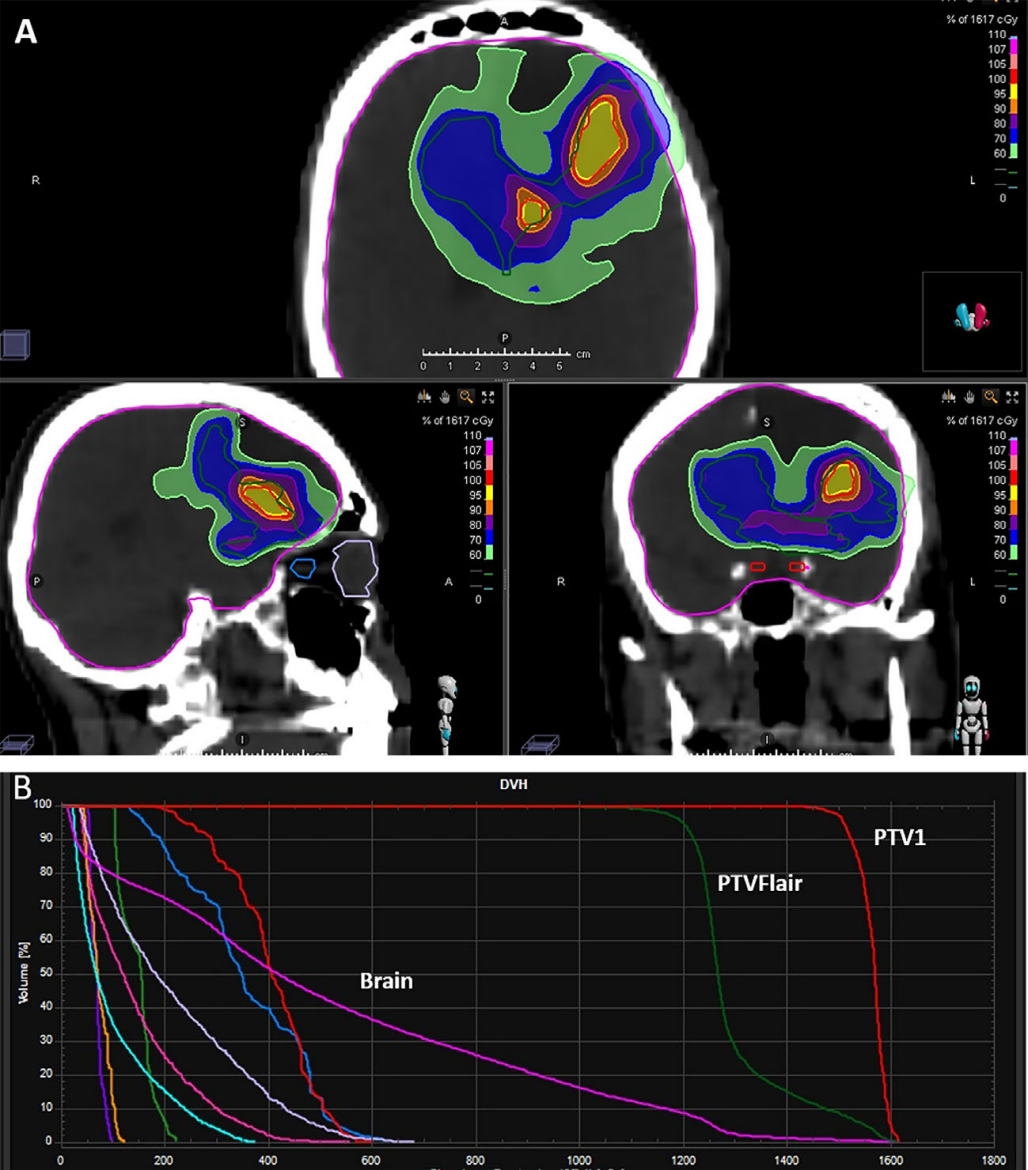
Examples of **(A)** dose distribution and **(B)** typical dose volume histogram (DVH) for a prescription dose of 15 Gy in 3 fractions to PTV1 and 12 Gy in 3 fractions to PTV FLAIR.

In patients in whom PTV FLAIR was delineated, a total dose of 12 and 20 Gy was delivered (99% isodose line covering 99% of the PTV); 15 Gy and 25 Gy were prescribed to the PTV1 in 3-5 daily fractions at the isodose of 67% (**Figure 1**). The treatment dose was chosen on the basis of the Karnofsky Performance Status (KPS) of the patient, the interval between the first and second radiotherapy course, tumour size, and the proximity of critical organs to the targets. All patients underwent image-guided helical TomoTherapy (HT) (TomoTherapy Inc., Madison, WI, USA). The HT system uses image-guided RT in which a CT scan is performed before each treatment, allowing the radiotherapist to verify and adjust the patient’s position as needed to ensure that the radiation is directed exactly at the target area.

#### Assessment of Response and Toxicity

The assessment of radiological and clinical response was based on MRI sequences obtained before and after HBO-RT according to RANO Criteria. The radiological protocol consisted in MRI T1-weighted imaging, contrast-enhanced T1-weighted axial imaging with gadolinium (Gd-MRI), fluid-attenuated inversion recovery imaging (FLAIR), axial T2- weighted imaging, DWI (diffusion-weighted imaging), dynamic susceptibility contrast-enhanced (DSC) and dynamic contrast-enhanced (DCE) perfusion. Each patient underwent MRI evaluation, neurological examination and Mini-Mental State examination (MMSE) 40 days after the end of RT and every 3 months thereafter for one year. Patients were followed until disease progression or death. All toxicities were recorded and graded according to NCI CTCAE (National Cancer Institute Common Toxicity Criteria for Adverse Events), version 4.3 (34).

### Evaluation and Statistical Analysis

Simon’s two-stage design was used to estimate sample size (35). In the first stage, nine patients would be accrued. If there were 3 or fewer DCRs in these 9 patients, the study would be stopped. Otherwise, 15 additional patients would be accrued for a total of 24. The null hypothesis would be rejected if 13 or more patients with DCR were observed in 24 patients. This design yielded a type I error rate of 0.05 and power of 80% for a true DCR of 0.62. The percentage of patients who achieved complete response, partial response and stable disease were calculated to evaluate the primary endpoint, and 95% confidence intervals (95%CI) were derived from the exact binominal distribution. For the safety assessment, the number and percentage of treated patients experiencing grades 1-4 adverse events were tabulated. OS and PFS were estimated with the Kaplan-Meier Method (two-sided 95%CI) and the role of potential stratification factors was analysed with the log-rank test.

## Results

### Patient Characteristics

Nine patients (2 females and 7 males) with rHGG were enrolled in this trial between February 2018 and October 2019. At time of the initial diagnosis, 7 (77.7%) had GBM, one had anaplastic oligodendroglioma (AO) and one had anaplastic astrocytoma (AA). The median age at the time of HBO-RT was 58.8 years (range 35.8-71.7 years). All patients had a Karnofsky Performance status (KPS) of ≥60. The entire cohort received adjuvant primary radiation therapy with concomitant chemotherapy after primary surgery. Eight patients underwent post-operative fractionated RT with a total dose of 60 Gy in 30 fractions and one received adjuvant hypofractionated RT with a total dose of 25 Gy delivered in 5 fractions. The median interval between primary RT and salvage RT was 17.2 months (range 4.3-23.5 months).The site of recurrence included 4 frontal lobe, 2 peritrigonal region, 1 temporal lobe, 1 hippocampus, 1 parietal lobe. The prognostic factor classes established by Carson et al. were applied to all patients (36). Specific patient characteristics are reported in **Table 2** and in **Supplementary Table 1**.

**Table 2:** Patient characteristics.

	No. (%)
**Median age at start of HBO-RT therapy (range)**	58.8 (35.8-71.7)
**Gender**	
Female	2 (22.2)
Male	7 (77.8)
**KPS**	
65	1 (11.1)
80	2 (22.2)
90	4 (44.5)
100	2 (22.2)
**Carson RPA classes**	
1	1 (11.11)
2	1(11.11)
4	1 (11.11)
6	5 (55.5)
7	1 (11.11)
**Post-operative RT (no. patients)**	
2 Gy daily (total dose 60 Gy)	8 (88.88)
5 Gy daily(total dose 25 Gy)	1 (11.12)
**Median interval between post-operative radiotherapy and salvage HBO-RT,** months [range]	17.2 [4.3-23.5]
**Salvage therapy before HBO-RT**	
None	9 (100)

HBO, hyperbaric oxygenation; Carson RPA, Carson recursive partitioning analysis; RT, radiotherapy.

### Treatment Delivered

All 9 patients completed RE-RT after HBO without interruption. Five patients underwent HBO-RT treatment over 3 consecutive days and the remaining four over 5 days (**Figure 1**). Details of the RT planning are reported in **Table 3**. The median time between HBO and the radiotherapy fraction was 24 minutes (04-50 minutes).

**Table 3:** Treatment details of HRT.

Patient	Site of recurrence	No. fractions	PTV1 (cc)	PTV FLAIR (cc)	Dose prescription to PTV1 (cGy)	Dose prescription to PTV FLAIR (cGy)	D1 (cGy) (PTV1)	D1 (cGy) (PTV FLAIR)
1	Right frontal	3	9.60	133.74	1500	1200	2401	2286
2	Left parietal	5	7.01	–	2500	–	3970	–
3	Left peritrigonal	5	7.26	59.62	2500	2000	3891	3891
4	Left frontal	3	12.40	–	1500	–	2437	–
5	Left temporal	3	23.08	–	1500	–	1556	–
6	Left frontal	3	5.57	94.46	1500	1200	1614	1589
7	Left peritrigonal	5	0.94	34.94	2500	2000	3876	3552
8	Left hippocampus	3	6.51	–	1500	–	2279	
9	Right frontal	5	5.96	50.73	2500	2000	3881	3253

HSRT, Hypofractionated Stereotactic Radiotherapy; PTV, planning target volume; FLAIR, fluid-attenuated inversion recovery; cGy, centigray; D1, dose to 1% of the volume.

### Outcomes

**Figure 2:**
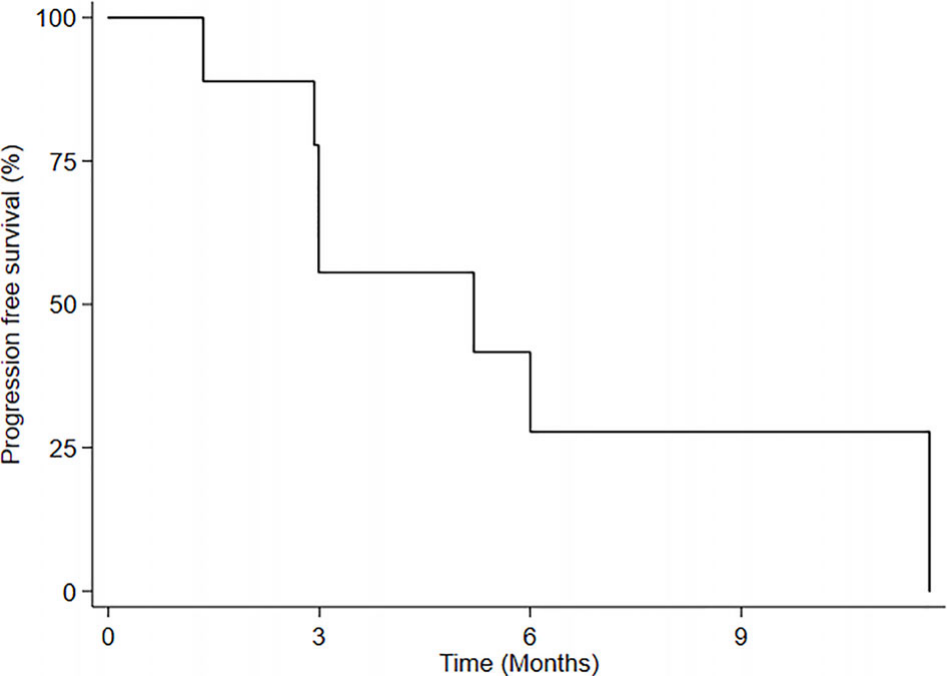
Progression-free survival (PFS) after HBO-RT.

Median follow-up from RE-RT was 11.6 months (range 3.2-11.6 months). No patient was lost to follow-up. Three months after treatment, 5 patients (55.5%) maintained local disease control, while 4 showed progression and the accrual of the first stage of the two-stage design was completed. Median progression-free survival (mPFS) for all patients was 5.2 months (95%CI: 1.34-NE) (**Figure 2**), while 3-month and 6-month PFS was 55.5% (95%CI: 20.4-80.4) and 27.7% (95%CI: 4.4-59.1), respectively. Upon progression, 2 patients underwent treatment with temozolomide, one with fotemustine and one with PCV (procarbazine, lomustine and vincristine).

**Figure 3:**
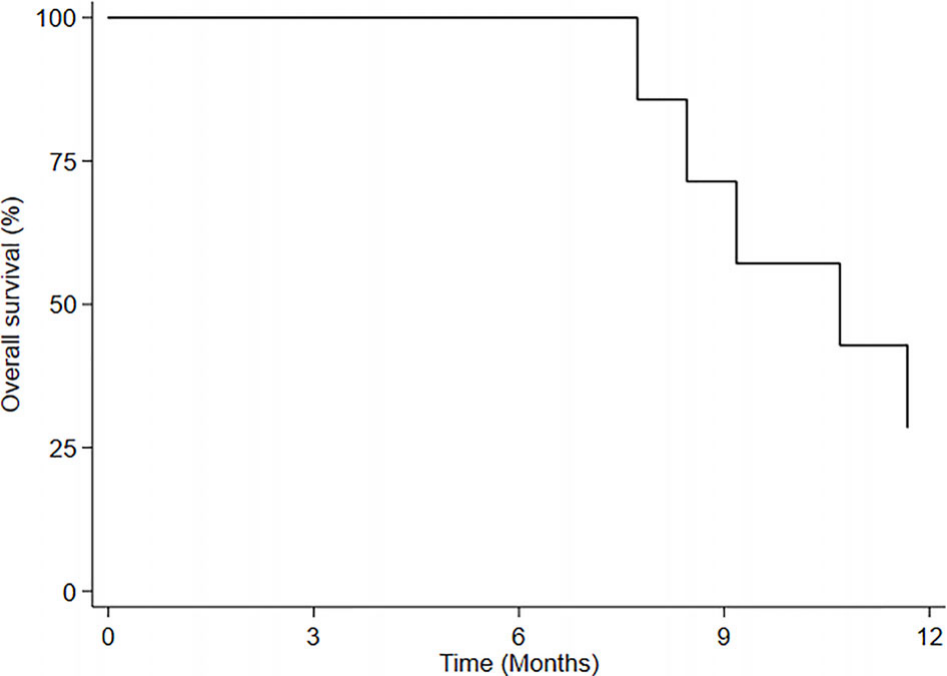
Overall survival (OS) after HBO-RT.

Median overall survival (mOS) of HBO-RT was 10.7 months (95% CI: 7.7-NE) (**Figure 3**). At time of this analysis, 5 patients with recurrent GBM (rGBM) had died (disease progression) and 4 were still alive, all living virtually normal daily lives until PD. Of this group, a 60-year-old woman obtained local disease control (3 months after HBO-RT); a 36-year-old man with recurrent GBM developed PD 12 months after completing HBO-RT and underwent treatment with bevacizumab; one patient with recurrent anaplastic oligodendroglioma progressed after 6 months; and one patient with recurrent AA progressed after 3months. Neurocognitive functions remained stable until PD. MMSE values for each patient were stable until PD.

### Toxicity

During HBO, only one patient experienced ear pain, without barotrauma. No patients had convulsive seizures during or after HBO. RE-RT was well tolerated. All patients completed treatment without interruption. During treatment dexamethasone ≥2 mg was administered to all patients. Three months after HBO-RT treatment Three patients continued with 2 mg dexamethasone while two took 4 mg dexamethasone and one 8 mg and three none. No acute or late neurologic toxicity >grade 2 (CTCAE version 4.3) was observed in 8 patients. A 71-year-old man with rGBM showed symptoms and radiological signs of grade 3 radionecrosis. When first diagnosed, the patient had GBM with unmethylated MGMT, negative IDH1 and IDH2, Mib1 10%, and positive GFAP. He underwent re-irradiation 24 months after postoperative RT, with a total dose of 25 Gy in 5 fractions to PTV1 (volume 0.96 cc) and 20 Gy in 5 sessions to PTV FLAIR (volume 34.94 cc). During follow-up, Gd-MRI, DWI, DSC and DCE perfusion MRI revealed a suspicion of radionecrosis and concomitant O-(2-[^18^F]fluoroethyl-)-L-tyrosine (^18^F-FET) PET/CT was performed to support the differential diagnosis of PD or treatment-related changes. The scans confirmed the radionecrosis. The patient was treated successfully with corticosteroids and bevacizumab.

## Discussion

The survival of patients with HGG depends on local disease control because the majority of patients die of recurrence at close proximity to the site of the primary tumour (37). Life expectancy after relapse is poor, and there is still no standard treatment for recurrent HGG, highlighting the need to develop effective salvage treatments to prolong OS (7). Several studies have suggested that re-irradiation may be a useful option for recurrent HGG, with acceptable toxicity (38). In fact, the availability of high-precision radiotherapy techniques permits retreatment, which is generally performed with single-fraction stereotactic radiosurgery, fractionated stereotactic RT (FSRT) or HSRT alone or in combination with systemic chemotherapy (39). The present paper reports the results of the first phase of a clinical trial, conducted according to a Simon’s two-stage design, to evaluate whether re-irradiation of rHGG after HBO can improve the efficacy of RT.

Failure of RT in malignant gliomas is primarily due to the presence of hypoxic, intrinsically radioresistant, cells in the lesion. In the early 1950s, Gray et al. postulated that oxygen deficiency was a main source of radiation resistance (40). The biological effect of ionizing radiation has been reported to be around 3-fold higher when it is delivered under well-oxygenated rather than anoxic conditions (40). Overgaard et al. (41, 42) studied various hypoxic modification techniques, reporting that HBO showed the most pronounced effect and could thus potentially improve RT results. Bennett et al. (43) recently reported that HBO may increase the effectiveness of RT in patients with head and neck cancer, reducing tumour regrowth and improving survival. HBO is based on the administration of 100% oxygen at higher than normal atmospheric pressure. It increases O_2_ tissue delivery independently of haemoglobin levels (44). Several authors have reported that the increase in tumour oxygen pressure is preserved for several minutes after HBO exposure. Kinoshita et al. monitored changes in MRI signal intensity after HBO exposure using non-invasive MRI. The authors demonstrated that the signal change related to the oxygen tension in murine squamous cell carcinoma VII (SCCVII) tumours decreased rapidly in the muscle after HBO but slowly in the tumour mass, and was still high 60 minutes after decompression (27). Beppu et al. stereotactically measured pO2 in both peritumoural and intratumoural glioma tissue after HBO, reporting significantly increased pO2 levels that remained stable for up to 15 minutes in both regions (17). Like Kinoshita et al., we estimated a maximum interval of 60 minutes between decompression and HSRT. Koshi et al. suggested that the timing of irradiation is vital to the overall success of RT following HBO exposure. In their study of the retreatment of high-grade gliomas, gamma FSRT was started within 7 minutes of the end of HBOT and lasted a full 80 minutes. In our study, the overall treatment time of TomoTherapy plans was much shorter, around 10 minutes (32). Al-Waili et al. showed that a combination of HBO and RT reduced tumour growth and improved local control, resulting in increased survival (45). Several studies have shown the feasibility of this treatment regimen in primary HGG, suggesting that HBO improves response rates and survival without serious side-effects in patients treated with RT (46–49).

Ogawa et al. treated 57 HGG patients with RT immediately after HBO, reporting a 52% objective response rate (47). Kohshi et al. used radiotherapy after HBO in HGG patients with residual disease, registering a 50% reduction in tumour mass and a median survival of 24 months (48). Yahara et al. evaluated the feasibility and efficacy of RT using an intensity-modulated radiotherapy (IMRT) boost after HBO together with chemotherapy in glioblastoma patients, reporting a median OS of 22 months (49). Only one study has been carried out on recurrent HGG patients treated with FSRT immediately after HBO (32). The authors, Kohshi et al., treated 25 patients with a median total dose of 22 Gy (range 18-27 Gy) in 8 fractions delivered to the tumour margin (32). They confirmed a survival benefit from this treatment, with low toxicity.

In our study, the disease control rate (DCR) 3 months after HBO-RT was 55.5% (5 patients), fulfilling the primary objective of the study and enabling us to open the second phase of recruitment. Median progression-free survival (mPFS) for all patients was 5.2 months (95%CI: 1.34-NE), while 3-month and 6-month PFS was 55.5% (95%CI: 20.4-80.4) and 27.7% (95%CI: 4.4-59.1), respectively. Median overall survival (mOS) of HBO-RT was 10.7 months (95% CI: 7.7-NE). These preliminary results are similar to those of other HSRT re-irradiation studies, i.e. PFS ranged from 4 months to 7.9 months and OS from 7.5 months to 11 months (**Table 4**).

**Table 4:** Studies on HSRT for recurrent high-grade glioma.

Author	No. patients	Median tumour volume	Median total dose (Gy)	Dose per fraction (Gy)	Median no. fractions	Bed_10_	Associated systemic therapy	Median PFS (m)	Median OS (m)
Vordemark et al. (50)	19	15	30	5	6	48	–	4.9	9.3
Ernst-Stecken et al. (51)	15	22.4	35	7	5	59.50	–	75% at 6 m 53% at 12 m	12
Fokas et al. (52)	53	35	30	3	10	39	–	22% at 12 m	9
Kim et al. (53)	8	69.5	25	5	5	37.50	–	4.6	7.6
Minniti et al. (54)	54	9.7	30	6	5	48	Tmz	6	12.4
Shapiro et al. (55)	24	35.3	30	6	5	48	Beva	7.5	12
Yazici et al. (56)	37	24	30	6	5	48		7.9	10.6
Minniti et al. (57)	54	12.4	25	5	5	37.50	Beva Fote	6 4	11 8.3
Navarria et al. (58)	25	35	25	5	5	37.50	Tmz Fotemustine Beva	16	18
Combs et al. (59)	325	54.4	36	2.67	20	42.48	Different regimens	–	7.5 (IV grade) 9.5 (III grade)

Beva, bevacizumab; Fote, fotemustine; TMZ, temozolomide; m, months.

Our pilot study consisted of HSRT after daily HBO for rHGG. The prescription doses delivered to PTV1 were 15 Gy in 3 fractions (5 patients), 25 Gy in 5 fractions (4 patients), and 12 Gy-20Gy to the PTV FLAIR in patient in whom PTV FLAIR was delineated. The equivalent dose in 2 Gy per fraction (EQD2) with alpha/beta 10 of 15 Gy in 3 fractions was 18.75 Gy, with a biologically effective dose (BED_10_) of 22.50 Gy_10_. The EQD2 of 25 Gy in 5 fractions was 31.25 Gy_2_, with a BED of 37.50 Gy_10_. Very different radiotherapy regimens were used in other HSRT re-irradiation studies, with fraction sizes ranging from 3 to 7 Gy and the number of fractions varying from 5 to 10 (**Table 4**) (50–62). Yazici et al. treated 37 patients with recurrent glioblastoma with a median dose of 30 Gy in a median 5 fractions (1-5 fractions) with a median volume of 24 cc (range 2-81). The authors reported a mPFS of 7.9 months and a median OS of 10.6 months (1.1-20 months) (56). The BED_10_ calculated for 30 Gy in 5 fractions was 48 Gy_10._ Minniti et al. delivered 25 Gy in 5 fractions in association with bevacizumab or fotemustine. Median PFS was 4 months for patients treated with HSRT plus fotemustine, and 6 months for HSRT and bevacizumab, with a median OS of 11 months (57). The BED_10_ calculated for 25 Gy in 5 fractions was 37.50 Gy_10._

In a recent multicentre study on re-irradiation of recurrent glioma, Navarria et al. identified a BED_10_ threshold of >43 Gy that influenced survival (60). Although our calculated BED is lower than that of other series, in particular that of 22.50 Gy_10_, our patients showed similar outcomes to those of other studies. Our ad interim analysis thus suggests a possible advantage of adding HBO to HSRT for the local control of rHGG.

Bennet et al. suggested that the dose per fraction may influence the importance of the benefit derived from hypoxic modification. They concluded that the use of hypofractionation results in a more pronounced modification of hypoxia (43). In our case series, we also used altered fractionation i.e. hypofractionation delivered by image-guided helical TomoTherapy, which enables large tumour volumes to be treated, minimizing the toxicity associated with high dose fractionation.

Our analysis ad interim showed only one case of radionecrosis grade 3 CTCAE.

Although the results from the present study suggest that the use of RT after HBO is a safe and practical procedure, our preliminary findings must obviously be interpreted with caution because of the small number and inhomogeneity of the patients involved. We thus aim to validate the results in the second part of the study in which another 15 patients will be recruited.

In conclusion, HBO-RT could represent an alternative, with low toxicity, to systemic therapies for patients who cannot or refuse to undergo such treatments. One of advantages of HBO- RT is the reduced overall treatment time (3-5 consecutive days). Further randomized studies in primary and recurrent settings are needed to confirm our findings.

## Data Availability Statement

The original contributions presented in the study are included in the article/**Supplementary Material**. Further inquiries can be directed to the corresponding author.

## Ethics Statement

The studies involving human participants were reviewed and approved by IRCCS IRST Ethics Committee. The patients/participants provided their written informed consent to participate in this study.

## Author Contributions

DA, EP, GG, PL, and PC conceived the idea for and designed the study. DA, MP, LT, EN, SM, MM, CA, FG, AC, and AS collected and assembled the study data. DA, EP, GG, PL, AT, and PC analysed and interpreted the data. FF performed the statistical analysis. DA drafted the manuscript. AR and GM revised the manuscript for important intellectual content. All authors contributed to the article and approved the submitted version.

## Conflict of Interest

The authors declare that the research was conducted in the absence of any commercial or financial relationships that could be construed as a potential conflict of interest.

## Abbreviations

AA, anaplastic astrocytoma; AO, anaplastic oligodendroglioma; BED, biologically effective dose; CT, computed tomography; DCE, dynamic contrast-enhanced; DCE, dynamic contrast-enhanced; DCR, disease control rate; DCR, disease control rate; DSC, dynamic susceptibility contrast-enhanced; DSC, dynamic susceptibility contrast-enhanced; DWI, diffusion-weighted imaging; DWI, diffusion-weighted imaging; *Fi*O2, fraction of inspired oxygen; FLAIR, fluid-attenuated inversion recovery imaging; Gd-MRI, axial imaging with gadolinium; HBO, hyperbaric oxygen therapy; HGG, high-grade glioma; HSRT, hypofractionated stereotactic radiotherapy; HSRT, hypofractionated stereotactic radiotherapy; HT, helical TomoTherapy; IMRT, intensity-modulated radiotherapy; KPS, Karnofsky performance status; MMSE, mini-mental state examination; mOS median overall survival; mPFS, median progression-free survival; MRI, magnetic resonance imaging; OAR, organs-at-risk; OS, overall survival; PFS, progression-free survival; PO2, pressure of oxygen; PTV, planning treatment volume; RANO, response assessment for neuro-oncology; RE-RT, re-irradiation; rHGG, recurrent HGG; rHGGs, recurrent malignant high-grade glioma; RT, radiotherapy.
