# Supplementary material for: Role of Hyperbaric Oxygenation Plus Hypofractionated Stereotactic Radiotherapy in Recurrent High-Grade Glioma
Source: Front Oncol. 2021 Mar 30;11:643469. doi: 10.3389/fonc.2021.643469 (PMC8042328; doi:10.3389/fonc.2021.643469)
Supplement: Supplementary file 1 [file Table_1.docx]

**Supplementary Table S1 Raw data**

| **Resection** | **Histology** | **MGMT** | **IDH1 mutation** | **IDH2 mutation** | **1p19q** | **Adjuvant primary therapy** | **Total**  **RT dose (Gy)** | **Adjuvant**  **chemo therapy** | **Salvage HBO-RT** | **MRI after 3 months** | **MRI**  **after 6 months** | **MRI after 9 months** | **MRI after 12 months** | **Treatment upon**  **progression**  **after HBO-RT** | **Date of death** |
| --- | --- | --- | --- | --- | --- | --- | --- | --- | --- | --- | --- | --- | --- | --- | --- |
| STR | OA | Unknown | Positive | Negative | Positive | RT | 25 | TMZ | 01/03/2018 | SD | PD | - | - | PCV | - |
| GTR | GBM | Positive | Negative | Negative |  | RT/TMZ | 60 | TMZ | 25/05/2018 | SD | PD | - | - | - | 02/02/2019 |
| STR | GBM | Negative | Negative | Negative |  | RT/TMZ | 60 | TMZ | 29/06/2018 | PD | - | - | - | FOTE | 31/03/2019 |
| BIOPSY | GBM | Unknown | Negative |  |  | RT/TMZ | 60 | TMZ | 26/07/2018 | PD | - | - | - | TMZ | 14/07/2019 |
| STR | GBM | Negative | Negative | Negative |  | RT/TMZ | 60 | NONE | 24/08/2018 | PD | - |  | - | TMZ | 13/07/2019 |
| GTR | GBM | Positive | Positive |  |  | RT/TMZ | 60 | TMZ | 24/08/2018 | SD | SD | SD | PD | BEVA | - |
| GTR | GBM | Negative | Negative |  |  | RT/TMZ | 60 | TMZ | 31/08/2018 | SD | SD | - |  | - | 19/04/2019 |
| STR | AA | Negative | Negative |  |  | RT/TMZ | 60 | TMZ | 28/08/2019 | PD | - | - |  | - | - |
| GTR | GBM | Positive | Positive |  |  | RT/TMZ | 60 | NONE | 11/10/2019 | SD | - | - |  | - | - |

STR, subtotal resection; GTR,gross tumor resection; OA, anaplastic oligodendroglioma; GBM, glioblastoma multiforme; AA, anaplastic astrocitoma; RT, radiotherapy; TMZ, temozolomide; SD, stable disease; PD, progressive disease; PCV, procarbazine, lomustine and vincristine; FOTE, fotemustine.
